# Implementation study of an interprofessional medication adherence program for HIV patients in Switzerland: quantitative and qualitative implementation results

**DOI:** 10.1186/s12913-018-3641-5

**Published:** 2018-11-20

**Authors:** Mélanie Lelubre, Olivier Clerc, Marielle Grosjean, Karim Amighi, Carine De Vriese, Olivier Bugnon, Marie-Paule Schneider

**Affiliations:** 1 Community pharmacy, School of Pharmaceutical Sciences, University of Geneva, University of Lausanne, Geneva, Switzerland; 2 Community pharmacy, Department of ambulatory care and community medicine, University of Lausanne, Lausanne, Switzerland; 3 Department of Pharmacotherapy and Pharmaceutics, Faculté de Pharmacie, Université libre de Bruxelles (ULB), Brussels, Belgium; 4 Department of Internal Medicine and Infectious Diseases, Pourtalès Hospital, Neuchâtel, Switzerland

**Keywords:** Implementation, Implementation outcomes, Re-AIM, Medication adherence, IMAP, Community pharmacy service, Interprofessional collaboration

## Abstract

**Background:**

An interprofessional medication adherence program (IMAP) for chronic patients was developed and successfully implemented in the community pharmacy of the Department of ambulatory care and community medicine (Lausanne, Switzerland). This study assesses the capacity of a physician and a nurse at the infectious diseases service of a public hospital and of community pharmacists in the Neuchâtel area (Switzerland) to implement the IMAP in their practice.

**Methods:**

Mixed method, prospective, observational study. Quantitative and qualitative analyses of the implementation process were conducted following the RE-AIM model (reach, effectiveness, adoption, implementation and maintenance).

**Results:**

Implementation started in November 2014. One physician, one nurse, and five pharmacists agreed to participate. Healthcare professionals perceived the benefits of the program and were motivated to implement it in their practice (adoption). Seventeen patients were included in the program; 13 refused to participate. The inclusion of naïve HIV patients was easier than the inclusion of experienced patients with difficult psychosocial issues (reach). Pharmacists were engaged in reinforcing patient medication adherence in 25% of interviews (effectiveness). Key facilitators expressed by healthcare professionals were patient inclusion by the physician and the nurse instead of the pharmacist and the organisation of regular meetings between all stakeholders. In contrast, the encountered barriers were the lack of time and resources, the lack of team uptake, and the lack of adoption by senior managers (implementation). Interviewed patients were all satisfied with this new program, encouraging healthcare professionals to scale it up. Structural changes allowed the hospital and one pharmacy to enter the maintenance stage (maintenance).

**Conclusion:**

The research team and collaboration between all professionals involved played an important role in this implementation. However, the dissemination of such a program to a larger scale and for the long term requires financial and structural resources as well as transitional external support.

**Electronic supplementary material:**

The online version of this article (10.1186/s12913-018-3641-5) contains supplementary material, which is available to authorized users.

## Background

Pharmacists are currently developing their role as a provider of professional pharmacy services through a number of various initiatives around the world [1]. In Switzerland, pharmacist interventions such as the polymedication check and medication adherence monitoring are remunerated by the healthcare system. However, even if a remuneration system exists, only a minority of pharmacists provide these interventions in practice and initiatives are often not implemented at scale across the population [2].

This trend is not confined to Switzerland; implementation of such services in practice remains difficult worldwide [3]. Recently, new studies adapted the consolidated framework for implementation research (CFIR) to list all potential factors that can influence the implementation of new services in community pharmacies [4, 5]. The most frequent barriers cited by pharmacists are lack of time, lack of resources/staff, lack of support/technical assistance, and inadequate infrastructure in the pharmacy [2, 3, 6–10]. The lack of motivation and the lack of benefits perceived by healthcare professionals are barriers that limit the adoption of these new services [2, 8, 11]. Moreover, external factors such as the patients’ needs and acceptance/perception, the lack of collaboration, and the opinion of other healthcare professionals also influence the implementation of new services [8, 10, 12, 13].

However, examples in the literature show that the implementation of community pharmacy services in practice is feasible [7, 14]. For example, the Community Pharmacy Center of the Department of Ambulatory Care & Community Medicine (PMU) of Lausanne (Switzerland) developed and successfully implemented an interprofessional medication adherence program (IMAP) for chronic patients [15]. The positive impact of this program has already been demonstrated for HIV patients. However, the capacity to implement it for HIV patients in other community pharmacies and medical settings has never been assessed [16]. To answer this question, an implementation evaluation study was conducted in 2014 during the implementation of IMAP in a public hospital “Hôpital neuchâtelois, Pourtalès” and community pharmacies in the Neuchâtel area (Switzerland). The main aim of this research was to evaluate the implementation outcomes of this project. The second aim was to collect patient clinical data, in particular medication adherence, viral load (VL), and CD4 cell count. A full description of the implementation stages and strategies is given elsewhere [17].

## Method

### Study design and framework

To evaluate the implementation outcomes at the same time as the collection of patient clinical data in this prospective observational study, we used the RE-AIM model with a mixed method approach [18]. This model was used to evaluate the transfer of the IMAP into the current health system and its effectiveness for the target population through five dimensions: reach, effectiveness, adoption, implementation (fidelity), and maintenance. Qualitative and quantitative results are presented by dimension and themes; results from the qualitative analysis are underlined with dotted lines. The mixed method approach, particularly the qualitative analysis, allowed us to describe the factors influencing the dimensions of interest. Considering the definition given by Curran et al., the study design is characterized as an hybrid type 3 design as the implementation outcomes were the first interest and clinical outcomes were collected for informative purposes [19].

### Description of the intervention and target patients

The IMAP combines motivational interviewing with feedback to the patient based on the medication adherence electronic measure (MEMS™, WestRock, Switzerland). The core content of the interview between the pharmacist and the patient is reported to the physician and the nurse. The development of the program is fully described elsewhere [15].

In this project, the target population are HIV patients whose medication adherence is not ritualized and/or patients who go through periods of suboptimal adherence. The physician and the nurse follow their patients to improve and/or stabilize clinical outcomes and medication adherence to prevent virological failure. Considering this aim, patient selection criteria for entering the IMAP were defined by the participating physician and nurse as either naïve HIV patients, patients with an antiretroviral switch or patients with psychosocial issues.

### Measured outcomes

The collected outcomes are described in Table 1. The clinical outcomes, e.g. VL, CD4 counts, and adherence data, were observed and described at the individual level as part of the maintenance dimension.

**Table 1:** Collected quantitative and qualitative outcomes

Dimension and definition [18]	Quantitative outcomes	Qualitative outcomes	Level of analysis
Reach = *The absolute number, proportion, and representativeness of individuals who are willing to participate in a given initiative*	• Reach rate: percentage of eligible patients who agree to participate (Inclusion/number of proposals*100)^a^		Hospital
	• Reasons for proposal		Hospital
	• Reasons for refusal		Hospital
	• Number of drop-outs		Pharmacies
	• Characteristics of the HIV population^b^		Hospital
		• Barriers and facilitators to including patients	HCPs and patients
		• Patients’ selection criteria	HCPs
Adoption = *The absolute number, proportion, and representativeness of settings and intervention agents who are willing to initiate a program*	• Adoption rate: pharmacists involved in the program among pharmacies of the Neuchâtel area (as a %age)		Pharmacies
	• Number of involved physicians/nurses in the hospital		Hospital
		• Perceived motivation for and utility of the program	HCPs
Implementation = *implementation refers to the intervention agents’ fidelity to the various elements of an intervention’s protocol*^c^	• Fidelity: number of delivered interviews, interval (in days) in between interviews		Pharmacies
	• Length of time for patient follow-up (in months)		Pharmacies
	• Time and cost needed to deliver the intervention		Pharmacies
		• Fidelity and adaptations^d^ (use of electronic monitors, motivational interviewing, and reporting)	HCPs and patients
		• Implementation barriers and facilitators	HCPs and patients
		• Interprofessional collaboration development	HCPs
Maintenance = *The extent to which a program or policy becomes institutionalized or part of the routine organizational practices and policies*	• Monitoring of patient inclusion		Hospital and pharmacies
		• Inclusion of the IMAP into the routine activity over time	HCPs
		• Satisfaction (professionals and patients)	HCPs and patients
Maintenance at the individual level = *the long-term effects of a program on outcomes 6 months or more after the most recent intervention contact*	• Adherence data		Pharmacies
	• VL and CD4 counts		Hospital

Notes: HCPs = Healthcare professionals = the physician, the nurse, and the five pharmacists included in the project

^a^Eligible patients were determined by the physician and the nurse and therefore considered as the number of proposals in this project

^b^Gender, age, ethnicity, education degree, source of HIV infection, antiretrovirals (ART) description at inclusion, ART line, VL, and CD4 count at inclusion

^c^In addition, implementation research has been expanded to include factors influencing the implementation process

^d^Information about fidelity and adaptations was collected by the research team through focus groups with HCPs and individual interviews with patients

### Data collection

Two research investigators (ML and MPS) collected the qualitative variables during semi-structured focus groups, organised every 6 months from April 2015 to December 2016. The interview grids were developed using the RE-AIM model and adapted over time according to the previous focus group results (Additional file 1). All themes discussed during these interviews are described in Additional file 2. During the first year, focus groups were organised with the physician and the nurse on the one hand and with pharmacists on the other. During the second year, they were brought together because of the positive atmosphere in the study groups and the willingness of healthcare professionals to increase their exchange of experience. If one pharmacist was absent during a focus group, an investigator (ML or MPS) visited this pharmacist for an individual interview and used the same interview grid as the one used during the focus group. Patients’ perceptions were also collected by an investigator during individual, semi-structured, taped phone interviews. Patient interviews were stopped after data saturation was reached.

Quantitative variables were collected from October 2014 to December 2016 in patient health records (hospital and pharmacy) and through the Swiss HIV Cohort Study (SHCS) database, an ongoing multicentre prospective observational study for interdisciplinary HIV-related research, which includes 69% of all HIV patients in Switzerland [20]. Variables were also collected prospectively during medical and adherence assessments at the hospital and at the pharmacies (SISPha™ Software) (see Table 1). To answer the question of the representativeness of included patients, we collected sociodemographic characteristics, VL, and CD4 counts for all HIV patients followed by the SHCS at the “Hôpital neuchâtelois, Pourtalès”. This data covered 1) included patients, 2) patients who refused the program, and 3) patients to whom the adherence program was not proposed because the program was not perceived as necessary by the physician and the nurse.

### Qualitative analysis

Individual and focus groups were transcribed verbatim identifying healthcare professionals and patients with numbers to ensure anonymity. Transcriptions were inductively coded by two independent investigators separately (ML and MPS), who then pooled the codes and discussed the discrepancies. Lastly, the codes were grouped into themes. To validate our data, the suggested themes were presented to all stakeholders.

### Statistical analysis

Descriptive data were represented as proportions for discrete variables and by the median and the interquartile range (IQR) for continuous variables, using Excel and STATA (StataCorp. 2013. Stata Statistical Software: Release 13. College Station, TX: StataCorp LP). Adherence data for the entire combined antiretroviral treatment (cART) were represented as the implementation dimension of adherence. Implementation of the dosing regimen is defined as the extent to which a patient’s actual dosing corresponds to the prescribed dosing regimen [21]. Patients were considered as adherent if this percentage was ≥90% [22].

## Results

The collaboration between the physician, the nurse, and the five pharmacists started in November 2014. All involved healthcare professionals were interviewed four times during the two-year study period. Eight patients (out of 17 included, 5 women – 3 men, median age 36 years [35–40]) were interviewed once. At least one patient per pharmacy was interviewed, which meant that all pharmacists were represented. All themes and related transcribed sentences are presented in Additional file 3.

### Reach

In 2 years, the program was proposed to 30 patients considered as eligible by the physician and the nurse; 17 agreed to participate, which represents a reach rate of 56.7%. The main reasons for proposal were a patient’s difficult psychosocial context that may affect medication adherence (*n* = 15), and the introduction of a new treatment, either a first or subsequent line (*n* = 9) (see Table 2). All 9 naïve HIV patients were included in the program with an acceptance rate of 100%, whereas patients with psychosocial issues tended to refuse the program more often (*n* = 10/15) (Table 2).

**Table 2:** Characteristics of the HIV population of the “Hôpital neuchâtelois-Pourtalès” between November 2014 and December 2016 (based on the patients’ medical records for included patients and the data of the Swiss HIV Cohort Study, SHCS, for patients who refused or were not proposed participation)

	Inclusion	Refusal	Not proposed
Number of patients	17	13	25
Gender (n, %)			
Male	10 (58.8%)	5 (38.5%)	15 (60.0%)
Female	6 (35.3%)	5 (38.5%)	10 (40.0%)
Transsexual	1 (5.9%)	0	0
Missing data	0	3 (23.1%)	0
Age (year) (median, IQR)	36 (31–42)	52 (39–56)	49 (41–54)
Ethnicity			
White	12 (70.6%)	9 (69.2%)	16 (64.0%)
Black	3 (17.6%)	1 (7.7%)	5 (20.0%)
Other ethnicity	2 (11.8%)	0	4 (16.0%)
Missing data	0	3 (23.1%)	0
Highest attained educational degree (n, %)			
Basic^a^	1 (5.9%)	1 (7.7%)	5 (20.0%)
Medium/high^b^	12 (70.6%)	9 (69.2%)	20 (80.0%)
Unknown/Missing data	4 (23.5%)	3 (23.1%)	0
Most likely source of HIV infection			
Sexual contact	13 (76.4%)	8 (61.5%)	22 (88.0%)
I.V. drug use/sexual contact (unclear which one)	2 (11.8%)	1 (7.7%)	3 (12.0%)
Other sources	2 (11.8%)	1 (7.7%)	0
Missing data	0	3 (23.1%)	0
ART description at inclusion^c^			
NRTI + NNRTI	7 (41.2%)	9 (69.2%)	15 (60.0%)
NRTI + INSTI	6 (35.3%)	0	2 (8.0%)
Other	3 (17.6%)	1 (7.7%)	5 (20.0%)
No treatment	0	0	3 (12.0%)
Missing data	1 (5.9%)	3 (23.1%)	0
Switch of treatment during the study (n, %)	7 (41.2%)	4 (30.8%)	6 (24.0%)
Treatment stop	0	1 (7.7%)	1 (4.0%)
Missing data	0	3 (23.1%)	0
ART line			
Naive patients^d^	9 (53.0%)	0	0
Experienced patients	8 (47.0%)	10 (76.9%)	24 (96.0%)
Missing data	0	3 (23.1%)	1 (4.0%)
VL at inclusion			
Patients with < 50 copies/mL (n, %)	7 (41.2%)	10 (76.9%)	24 (96.0%)
Patients with ≥50 copies/mL (n, %)	10 (58.8%)	0	1 (4.0%)
Missing data (n, %)	0	3 (23.1%)	0
CD4 at inclusion (median, IQR)			
CD4 count (cell/mm3)	583 (434–687)	753 (659.5–931.5)	648 (406.5–908.5)
Reasons for proposal (n, %)^e^			–
Difficult psychosocial context	5	10	
Introduction of a new ART	8	1	
Difficulties described by the patient	2	1	
Suspected non-adherence by health professionals	3	0	
Other	1	1	
Reasons for patient refusal (n, %)^e^	–		–
Refused to change pharmacy		11	
Doesn’t feel the need for such support		2	
Fears about confidentiality		2	

Notes:

^a^ Did not complete school or professional education, mandatory school

^b^ Finished apprenticeship, bachelor’s degree, higher professional education, higher technical or commercial school, university or other equivalent educational degree

^c^ NRTI = nucleoside reverse-transcriptase inhibitors, NNRTI = non-nucleoside reverse-transcriptase inhibitors, INSTI = integrase inhibitors

^d^ First ART started < 1 month ago

^e^ More than one reason was possible

The number of included patients per pharmacy was low (2 to 3 patients), except for the pharmacist located near the hospital, who followed 9 patients. The included patients were mostly men (*n* = 10; 58.8%) and younger than patients who refused (median age 36 [31–43]). No drop out was recorded in the study. Characteristics of the entire HIV population of the hospital are presented in Table 2.

### Facilitators for reaching patients

Facilitators for reaching patients were their inclusion by the physician and the nurse and specifically the inclusion of naïve HIV patients, to whom the program was presented as a package linked to the new treatment. Quantitative data confirmed this statement, as 100% of naïve HIV patients agreed to participate (see Table 2). According to interviewed patients, the physician and the nurse described the program appropriately and information received by patients was reassuring.

### Refusals and barriers to reaching patients

The main reported reason for refusal was the patient’s reluctance to change pharmacy, especially because of an existing trust relationship with the pharmacist (*n* = 11/13). However, the physician and the nurse explained that reasons were sometimes unclear, a possible escape for some complex patients who did not want to talk about their medication adherence. These patients are difficult to reach; they also tend to avoid talking with the nurse trained in therapeutic education at the hospital.

Different barriers have been encountered by healthcare professionals to reach patients. These can be HIV-related difficulties (psychosocial issues with stigmatisation, denial and need for a high confidentiality level) and the limited number of trained pharmacies, reducing the choice for patients. The small population base of HIV patients also explained the small number of included patients.

Another factor was non-inclusion by pharmacists who had difficulties in identifying target patients among their regular HIV patients and in proposing the program at the counter, which could breach the confidentiality needed by many HIV patients. Moreover, they had difficulties in acknowledging the added value and benefits of the program and were afraid to propose a “paid service” to patients, even if the program is supported by the Swiss national health insurance system.

### Adoption

#### Motivations of healthcare professionals

At the hospital, the initiating event for adopting the IMAP was a local experience with a non-adherent patient, whose need for medical and nursing time was disproportionate compared to the other patients. The physician and the nurse became aware of the need for additional support, which spurred them to contact the Lausanne community pharmacy to implement the IMAP in their practice. The hierarchy of the infectious disease service was informed of the program and its development.

In the Neuchâtel area, five community pharmacies were trained and ready to deliver the program, which represented 9% of Neuchâtel pharmacies (*n* = 5/56). Participating pharmacists were convinced that such a service is part of the future of the profession and perceived it as an opportunity to prove their added value. The IMAP, in collaboration with the hospital, was seen as a springboard and an opportunity to include patients and get more experience in medication adherence. For all healthcare professionals, motivations to integrate the IMAP in their practice were to support complex patients and to develop interprofessional collaborations.

#### Perceived utility of the program by healthcare professionals

All healthcare professionals agreed that the program was useful for patients. Firstly, they observed improvements in clinical results. Secondly, patients developed a relationship of trust with the pharmacist, who became a reference person for the patient at the pharmacy. The physician also noted that the pharmacist was a contact point for keeping patients in care and avoiding losing patients for follow-up. Thirdly, the use of electronic pillboxes seemed to reassure patients, allowing them to visualize their medication intake.

### Implementation

#### Time and cost

The total time needed at the hospital to deliver the program was 30 to 40 min per patient (proposal: 5–10 min, contact with the pharmacist: 15 min, discussion with the patients about medication adherence results during follow-up visit: 10–15 min). No specific costs were required at the hospital to implement the program. For the physician and the nurse, this new program took more time at the beginning of the implementation process, but is now integrated into a routine and does not require substantial additional time. For pharmacists, the program delivery took approximately 45 min for a first interview and 20 to 45 min per patient for a follow-up interview (interview preparation: 5 min, interview: 5–30 min, report (writing and sending): 10 min). The regular meetings, organised every 6 months between stakeholders, took approximately two hours. The rent of the meeting room was zero as the meetings took place in the hospital.

To implement the program in practice, pharmacists had to pay 4500 CHF per year for the web platform, which includes 5 days of training available for the pharmacist and one day for the pharmacy technician.

#### Intervention fidelity and adaptations

The intervention consisted of patient-pharmacist motivational interviews based on the medication adherence results of the electronic monitors, followed by a medication adherence report sent to the physician and the nurse. In total, 63 medication adherence interviews were encoded on the web platform by the pharmacists. The median interval in between interviews was 48 days [30–73] and the median follow-up time since inclusion was 1.7 years [0.6–1.9]. Two pharmacists (2/5) had a high fidelity to the program. They fully integrated the communication skills of motivational interviewing, they used the electronic monitors, and emailed reports on each interview to the physician and the nurse. The other three pharmacists balanced between a paternalistic and a shared responsibility attitude with patients. However, with time, most of them developed active-listening skills, progressed at the patient’s rhythm, looked for solutions in collaboration with patients, and valued patients’ self-management achievements. They connected fluctuations in medication adherence over time to living conditions, e.g. a drop in cART adherence during an overwhelming postpartum period. During the first year, pharmacists expressed fears about tackling some adherence issues with patients and thought that patients would perceive the intervention as a ‘policing’ one. This feeling decreased over time and pharmacists became more at ease with patients.

Only one pharmacist, who followed two patients, did not use the electronic monitors because neither the patients nor the pharmacist were at ease with the monitoring and perceived it as a form of control instead of a support.

Finally, we observed that pharmacists did not systematically send the adherence report to the physician and the nurse. Some pharmacists had concerns about the relevance of the transmitted information. Moreover, pharmacists had concerns about disturbing the physician with too many reports and increasing his workload. However, all pharmacists contacted the physician or the nurse in case of medication adherence problems. Even so, the physician had to express several times his need for regular medication adherence reports as this dimension matters and guides the medical patient’s follow-up, whether medication adherence is high or low. This repeated information motivated the pharmacists, who started sending the report more regularly.

The flow diagrams of the activity as described by the physician, the nurse, and community pharmacists are presented in Additional file 4. Compared to the original IMAP, pharmacies in the Neuchâtel area have not yet integrated pharmacy technicians into the program.

#### Implementation facilitators and barriers

The discriminant facilitator was the program’s involvement of the physician and nurse in including patients and in acknowledging the pharmacist’s role in supporting medication adherence. The second was the resources put in place for the program (research support, web platform, and trainings) and particularly the regular meetings between stakeholders organised by the research team. These meetings were an important step in driving the project forwards. They were described as rewarding and stimulating, and allowed healthcare professionals to get feedback from each other and exchange experience with their colleagues. These interprofessional contacts helped them to learn about each other’s expectations and meet the physician and the nurse face-to-face, which facilitated communication in practice. The physician and the nurse also raised the fact that the small size of the medical team facilitated internal communication on including patients at the beginning of the implementation project.

In contrast, the largest barrier encountered by healthcare professionals was a lack of time, related to lack of resources. For the physician and the nurse, the lack of time was a barrier to the program when a patient had somatic problems that had to be prioritized during the medical visit. For pharmacists, staff were already engaged in a full-time workload with the usual pharmacy activities. Another barrier encountered by pharmacists was the low adoption by pharmacy managers because of a lack of sufficient financial incentives. Some pharmacists also reported a lack of team uptake. For example, only one pharmacist was in charge of the program per pharmacy.

Patients described the time required for interviews as acceptable as they perceived the benefits of the intervention and they appreciated the organisation of interviews at the pharmacy. No barrier was encountered by patients.

#### Development of interprofessional collaboration

**Fig. 1:**
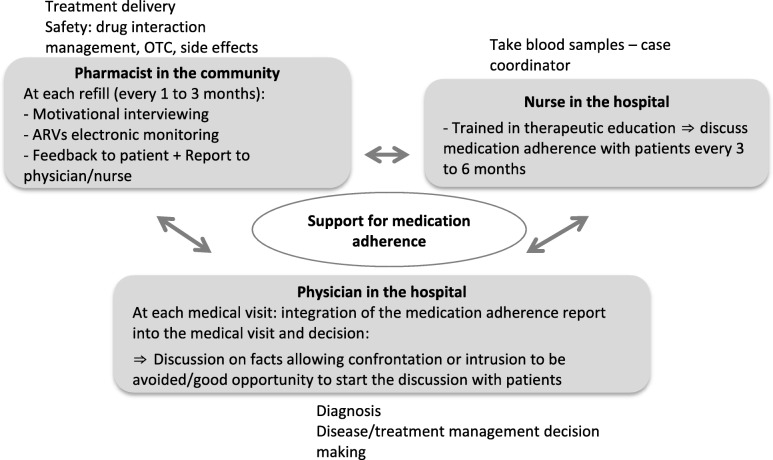
Respective roles of each healthcare professional in the IMAP. Note: ARVs = antiretrovirals

The respective roles of each healthcare professional in the program are described in Fig. 1. All professionals see patients at different intervals and are complementary to each other. Five practical benefits of this collaboration were perceived by healthcare professionals. The first was the synergy and the complementarity of the information given by the patient to each professional. Second, the pharmacist’s report was useful for the physician during the medical visit to open up discussion with patients on medication adherence and to integrate this information into clinical decisions. Third, the program was reassuring for the physician and the nurse as it strengthens patient safety regarding drug interaction management, OTCs, and regular treatment delivery. Fourth, the program was an opportunity to share responsibilities for complex patients, and fifth, an opportunity to decrease the risk of patient loss for follow-up.

### Maintenance

#### Monitoring of patient inclusion

**Fig. 2:**
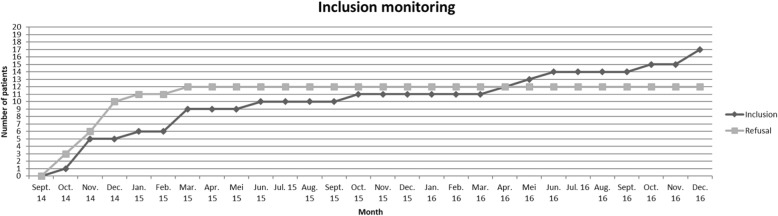
Patient inclusion monitoring during the two-year follow-up

Figure 2 shows that refusals occurred only during the first months of the project and revolved around experienced patients. Based on this initial experience, only new HIV patients or patients with a switch of treatment have since been included in the program by the physician and the nurse.

#### Inclusion of the program into the routine activity

In May 2016, a structural change at the hospital allowed all new interns on rotation to stay in the service longer, i.e. for 6 months. This change allowed the physician and the nurse to integrate the new intern in the program and lead the nurse to formalize the IMAP, including it in the local hospital guidelines. This step represented the transition to the maintenance stage.

Four pharmacists were in the operation stage at the end of the project. One pharmacy included a second pharmacist in the program and implemented a team organisation, with a shared calendar to organise interviews with patients. An increase in patient inclusions, including patients with other chronic diseases, e.g. diabetes, has been made possible with this new organisation. This brings the program into the pharmacy’s routine activity, and therefore the sustainability stage.

#### Healthcare professionals’ satisfaction

All healthcare professionals were satisfied with this new program, considering it as an ideal collaboration and a new professionally satisfying activity. The positive feedback and the acknowledgement received from patients reinforced their idea that the program improves patient support. Moreover, as reinforced by pharmacists, the project was instructive and opened doors to more pathologies, such as hepatitis C or type II diabetes.

#### Patient satisfaction

All interviewed patients (*n* = 8) had a positive perception of the program and were satisfied. They all experienced good collaboration with healthcare professionals and five patients stated that they had developed a relationship of trust with them.

Four patients expressed that the interview with the pharmacist represented additional social support and reassured them. Five patients also reported that it allowed them to benefit from a reference person at the pharmacy. Three patients perceived the interview with the pharmacist as a new opportunity to discuss their medication, whereas four stated that they received support in their treatment management. The four latter patients reported that they were more aware of the importance of the treatment intake and more active in the search for solutions in collaboration with healthcare professionals. Finally, one patient perceived the feedback from professionals as encouraging.

Concerning the electronic monitors, six patients stated that they were satisfied with the electronic monitor, which reassured them and prevented omissions through the real time LCD display.

#### Intervention outcomes

We observed that all 9 naïve HIV patients included in the program and one experienced HIV patient who was detectable at inclusion became undetectable during the follow-up. Only one blip, a temporary detectable increase in viremia (VL), was observed in a patient during the follow-up. Patients who refused the program were all undetectable at the moment of the proposal and stayed undetectable during the entire two-year study period. Two patients who were not invited to join the program had a blip during the study period. CD4 counts are presented in Table 3.

**Table 3:** Patient CD4 cells counts at inclusion and at the end of the study period

	CD4 counts^a^ (cell/mm3) (median, IQR) [95% CI]	
	At inclusion	At the end of the study period
Included patients (*n* = 10)		
Naïve patients (*n* = 3)	280 (272–374) [272–374]	524 (516–910) [516–910]
Experienced patients (*n* = 7)	654 (535–1167) [452–1170]	828 (751–882) [473–1043]
Patients who refused the program (*n* = 9)		
Experienced patients	743 (682–970) [642–1011]	836 (712–1031) [639–1232]
Patients who were not invited to join the program (*n* = 22)		
Experienced patients	697 (496–910) [496–910]	685.5 (502–851) [502–851]

Notes: This table refers to the available CD4 data in the SHCS and therefore does not represent all the hospital’s patients; ^a^ Paired data

Median adherence during the entire monitoring period was 99.5% [90–100] and was below 90% in 2 experienced HIV patients. Five patients had transient suboptimal adherence (< 90%) in a total of 13 inter-visit periods. This means that pharmacists were engaged in reinforcing patient medication adherence in 25% (*n* = 13/52) of registered interviews.

## Discussion

### Feasibility

This study showed that the physician and the nurse at the infectious disease service of the Neuchâtel hospital and local community pharmacists were able to implement the IMAP in their practice. The program was clearly adopted by healthcare professionals, who perceived the benefits of the program for the patient’s follow up and were motivated to implement it in their practice. The physician and the nurse were convinced of the positive role of the pharmacist in improving medication adherence and the regular meetings, which allowed real interprofessional collaboration to develop and become ingrained in practice. The interviewed patients were all satisfied, which encouraged healthcare professionals to continue to deliver the program. The external support of the research team and of SISPha was an important factor in driving the project forward.

Naïve HIV patients were easier to include. Experienced patients with psychosocial context, to whom the program was proposed as a preventive measure, more often refused to be included. The stigmatisation existing around patients with psychosocial troubles may have hindered their inclusion. These observations brought the physician and the nurse to keep an eye on patients who refused the program and to systematically propose the program to new naïve HIV patients.

The healthcare professionals’ motivation and the positive perception of the program were facilitators of this project, even though the lack of motivation and physician’s program perception are often described as barriers in the literature [2, 8, 11]. However, a lack of time and resources, frequently cited in the literature, were also described as barriers in this project, particularly by pharmacists [3]. The physician and the nurse cited the lack of time as a barrier but adapted their practice and included this new activity without major effort by formalizing their actions. Before the project, the physician and the nurse were aware of the non-adherence problem and already discussed it with patients during the medical visits. Moreover, the total time needed to deliver the program is lower for them than for pharmacists, which may explain why pharmacists encountered more difficulties in including it in their practice. Moreover, pharmacists faced a financial barrier as they had to develop the program without extra funding/resources. Indeed, most HIV patients only had one prescribed medicine formulation combining the entire tritherapy, whereas the Swiss national insurance system reimburses the pharmacist polypharmacy service if at least three medicine formulations are prescribed simultaneously.

To implement this program in their practice, pharmacists had to endorse a “new position” and had to integrate structurally the professional team who follow the patients. Pharmacists were not comfortable including HIV patients at the pharmacy because of confidentiality issues. Indeed, pharmacists were more at ease when patients were included by the physician and the nurse in a hierarchical structure as the program was then perceived by the patients as a medical prescription. Having newly joined the interprofessional team, pharmacists still doubted the credibility of their actions and the relevance of the transmitted information. These doubts resulted in irregular reporting of medication adherence to the physician and nurse. Through regular meetings, the physician and the nurse took the opportunity to reinforce their expectation of systematic medication adherence reports, whatever the patient adherence level (regular, stable, high vs. low, or decreasing). Consequently, pharmacists became more confident over time. These results show that the pharmacists’ nascent position in the team was strengthened by regular meetings and the active involvement of the physician and the nurse; both were seen as facilitators by pharmacists. These results can be highlighted by Bandura’s theory, which provides a conceptual framework for self-efficacy and explaining the link between social-structural factors and organizational performance [23]. In this study, the active involvement of the physician and the nurse (social persuasion), the exchange of experience with other pharmacists (vicarious experience), and feedback from patients (mastery experience) improved and reinforced pharmacists’ beliefs about their capacity to deliver the program over time.

### Maintenance and perspectives

In this implementation, the systematization of actions and the uptake of the team were important to envision the maintenance of the program in a long-term perspective. This is aligned with the literature [10, 12]. Despite the encountered barriers, healthcare professionals continued to deliver the program and wanted to extend it to other pathologies. Pharmacists considered it as part of the future of their profession, but the financial barrier, which led to a lack of resources and time, may hinder the long-term development of the program and its development to a larger scale. Future strategies should be developed to maintain the program in place, including the improvement of the uptake by the team and workload organisation. The dissemination of the program and important strategies, such as organising regular meetings, were described and discussed in another article describing the implementation process of the project [24].

### Strengths and limitations

This two-year implementation study that brought information on maintenance has some limitations. Firstly, the number of encoded medication adherence interviews was probably underestimated as pharmacists were not consistent in data recording and the reporting. Secondly, the fidelity of the intervention in motivational interviewing was evaluated both through focus groups with healthcare professionals and in individual interviews with patients, which allowed us to cross check the qualitative data and understand how interviews were delivered. We used this method to minimize the bias of self-reporting by healthcare professionals regarding their practice. The fidelity regarding motivational interviewing should be further assessed through an additional assessment method (e.g. continuous training and timely supervision according to the recommendations of the motivational interviewing group [25]). Thirdly, interviewed patients were all satisfied but we cannot exclude a reporting bias, even though research and not clinical staff performed the interview and the anonymity of the answers was guaranteed. Fourthly, the low number of included patients made the assessment of the intervention’s effectiveness impossible. Nevertheless, medication adherence was suboptimal in 25% inter-visit interviews, giving frequent opportunities to pharmacists in the community to discuss and improve medication adherence issues in collaboration with the patients on a timely basis. Patient inclusion will continue with the maintenance of the program, allowing us to assess its effectiveness in the future. Fifthly, regarding the selection of target patients, all naïve HIV patients were included but some complex experienced patients were more complicated to include. In such patients, a treatment switch or a detectable viral load could be timely facilitators to leverage their inclusion.

## Conclusion

The IMAP is a replicable model that can be adapted to other settings. This program is transposable to all chronic patients for whom medication adherence is not ritualized and/or who are going through difficult adherence periods. The active involvement of the physician and the nurse and the organisation of regular meetings between all stakeholders helped to drive the project forwards and to significantly improve the interprofessional collaboration. However, the dissemination of such a program to a larger scale requires resources and external support. Finally, the monitoring of patient inclusion, the use of the electronic monitors and medication adherence reporting, and the interprofessional collaboration should be continuously assessed over time to confirm their maintenance beyond the first 2-year implementation.

## Additional files

Additional file 1: Interview grids developed for focus groups with health care professionals and individual interviews with patients. Description of data: Interview grids (translated version) used during focus groups with health care professionals and individual interviews with patients. (DOCX 44 kb)

Additional file 2: Content of organised meetings and focus groups during the operation phase. Description of data: Description of topics covered during meetings and focus groups with health care professionals. (DOCX 20 kb)

Additional file 3: Themes inferred during the qualitative analyses and associated sentences. Description of data: Themes defined by researchers and associated sentences classified by dimensions and measured outcomes. (DOCX 54 kb)

Additional file 4: Flow diagram of the IMAP as implemented at the hospital and in community pharmacies. Description of data: Implemented activity at the hospital and in community pharmacies as described by each included health care professional 2 years after implementation start (1 physician, 1 nurse, 4 pharmacists) (compiled version). (DOCX 73 kb)

## Abbreviations

IMAP: Interprofessional medication adherence program
PMU: [Policlinique Médicale Universitaire] = Department of ambulatory care and community medicine (Lausanne, Switzeland)
VL: Viral load
