# Supplementary material for: Implementation study of an interprofessional medication adherence program for HIV patients in Switzerland: quantitative and qualitative implementation results
Source: BMC Health Serv Res. 2018 Nov 20;18:874. doi: 10.1186/s12913-018-3641-5 (PMC6247756; doi:10.1186/s12913-018-3641-5)
Supplement: Supplementary file 1 — Interview grids developed for focus groups with health care professionals and individual interviews with patients. Description of data: Interview grids (translated version) used during focus groups with health care professionals and individual interviews with patients. (DOCX 44 kb) [file 12913_2018_3641_MOESM1_ESM.docx]

**Additional file 1 - Interview grids developed for focus groups with healthcare professionals and individual interviews with patients**

1. **First focus groups with healthcare professionals**
   1. *With the physician and the nurse*

| **Outcomes** | **Open-ended questions** |
| --- | --- |
| Motivation | What are the reasons that motivated you to integrate this medication adherence program for HIV patients in your practice? |
| Perceived utility | In your opinion, what is the utility of this program? Could you illustrate it with concrete examples? |
| Inclusion process | Who proposes the program to the patient? |
| Patient selection criteria | What are the patient selection criteria?  Have you sometimes chosen not to offer the program to an eligible patient and for what reasons? |
| Barriers | What are the difficulties you have encountered? |
| Facilitators | What factors have facilitated the implementation of the HIV patient medication adherence support program? |
| Time to deliver the intervention | How long does it take to introduce the program at inclusion?  How long does it take to debrief the report with your patients during the medical visit? |
| Integration into routine activity | How would you describe the integration of this program into your usual activity? How do you break down the work between the physician and the nurse/how do you disseminate information between you? |
| Interprofessional collaboration and communication with patient | What are your relationships with pharmacists and with patients? When do you contact pharmacists and why? |
| Positive or negative impact on patients | In your opinion, what is the impact of this follow-up on your patients? Could you give us specific examples? |
| Satisfaction | What are your impressions of the program and how it works? What are the benefits that result from it in your clinical activity? |
| Suggestions for improvement | What would your suggestions for improvement be regarding the program and its practical progress? |

- 1. *With pharmacists*

| **Outcomes** | **Open-ended questions** |
| --- | --- |
| Motivation | What are the reasons that motivated you to integrate this medication adherence program for HIV patients into your practice? |
| Perceived utility | In your opinion, what is the utility of this program? Could you illustrate it with concrete examples? |
| Barriers | What are the difficulties you have encountered? |
| Facilitators | What factors have facilitated the implementation of the HIV patient medication adherence support program? |
| Time to deliver the intervention | How much time did you spend on average per patient reading the electronic monitors, preparing for the interview, interviewing and writing the report? And on the exchange of information with the medical team and other partners? |
| Integration into routine activity | How would you describe the integration of this program into your usual activity? How do you break down the work between the pharmacist and the pharmacy technician? |
| Interprofessional collaboration and communication with patient | How would you describe your relationships with other health professionals and with patients? |
|  | What contacts do you have with the physician/nurse in this program? How do you send the reports to the physician/nurse? |
|  | How often do you meet patients who have joined this program? How do you establish this frequency of visit? In what circumstances did you have to contact your patients? |
| Positive or negative impact on patients | In your opinion, what is the impact of this follow-up on your patients? Could you give us specific examples? |
| Satisfaction | What are your impressions of the program and how it works? What are the benefits that result from it in your pharmaceutical activity? |
| Suggestions for improvement | What would your suggestions for improvement be regarding the program and its practical progress? |

1. **Second focus groups with healthcare professionals**
   1. *With the physician and the nurse*

| **Outcomes** | **Open-ended questions** |
| --- | --- |
| Integration into routine activity | What has been the evolution of the program integration in your routine activity over the last 6 months? How would you describe this integration? |
| Patient selection criteria (screening) | What are the patient selection criteria?  To what extent has the experience acquired in recent months influenced the screening of patients? (Evolution of these criteria over time)  Have you sometimes chosen not to offer the program to an eligible patient and for what reasons?  Why do you think patients refuse to participate in the program? How did you perceive these refusals? How do you consider them?  You told us about a patient reluctant to participate in the program and to whom you are talking about the program at every visit. Did you propose it to him again, and did he accept it?  Do you have other examples of patients like this? Have there been any reversals for patients (refusal <-> acceptance)? What lessons did you draw from these refusals? What should be done for those patients who refuse? |
| Proposal of the program | What leverage is used to present the program to patients? How can this proposal evolve over time? How is this proposal perceived by patients? Has there been a change in the response of patients? Is there a good/special time to talk to a patient about this program? Do these moments help you with screening? |
| Report | Once the patient is included, is the report debriefed with your patients? What elements of the report do you use for the patient? During the last focus group, you wanted to receive more reports from pharmacists. How is that going now? How often do you receive these reports? Is that okay for you? What feedback do you give to the pharmacist regarding the reports? |
| Facilitators | What new facilitators have you encountered in the last 6 months? |
| Satisfaction | How has your impressions of the program and its practical progress changed? What are the benefits that result from it in your clinical activity? |
| Barriers | Conversely, what are the frustrations and questions that arise in the context of your clinical activity? What new barriers have you encountered since our previous focus group? One of the barriers mentioned was the limited availability of participating pharmacists. Is this barrier still relevant today? How easy or difficult do you find it to reach the pharmacist in charge of the program? |
| Time to deliver the intervention | How has the time spent presenting the program and the contact with the pharmacist changed?  How much time do you spend debriefing the report with your patients? |
| Interprofessional collaboration and communication with patient | How have your relationships with pharmacists evolved? Apart from the patient inclusion, when do you contact pharmacists and for what reasons? |
| Positive or negative impact on patients | Recently, you told us about the improvement in clinical results for a patient. Could you give us other specific examples of clinical improvement? What other non-clinical impacts does this program have on your patients? What do you think this program is strengthening in patients, other than adherence?  Have you noticed a negative impact? If yes, what? How could you correct it? |
| Motivation for implementation and perceived utility | During the last focus group, you mentioned the following points as motivational elements, or elements in the usefulness of the program:   - Experience and training - Support for patients with psychosocial issues, support for medication adherence and additional space for dialogue - Interprofessionality: Additional support, additional reference person, proximity of the pharmacist.   What other points come to mind?  You told us about this patient, who was driving the project. Are there other examples that could illustrate the various points mentioned? |
| Personal involvement | How do you perceive this new program within your usual professional activity? Did you expect that? What is the structural/organizational impact of this new program?  What does this change mean to you? Overall, how do you position this new program within your usual professional activity? Has your personal involvement in the project evolved? How do you see it next? |
| Suggestions for improvement | To what extent does the proposed material (proposal sheet, presentation of the program) fit into your routine activity? You proposed to expand the program to hepatitis C patients. How has this proposal evolved? Is the material used for HIV patients also used for these patients? What are your other suggestions for improvement now? |

- 1. *With pharmacists*

| **Outcomes** | **Open-ended questions** |
| --- | --- |
| Fidelity and adaptations | How are the interviews conducted? The usual steps are that the patient arrives at the pharmacy, he has an appointment with you, you prepare the interview, you welcome him, the interview takes place, you document the intervention through SISPha and finally you send the report. How are these different steps going? *(What do you talk about with the patient? Do you have a particular structure?)* What has been the evolution of the integration of this program into your routine over these last 6 months?  We understood that the interviews took place according to prescription renewals. How has the frequency of interviews changed in the last 6 months? How is it going now? The pharmacy technicians were not integrated in the program. How is that now? Have you divided the work between the pharmacist and the pharmacy technician? What difference do you perceive between interviews at the counter and interviews in private? |
| Time to deliver the intervention (preparation, interview, report) | In April, you said that about 30 minutes were needed to deliver the intervention (5 minutes for preparation, 15-30 minutes for interview and 10-20 minutes for the report writing, often on down time). How this time has evolved over these last 6 months? What is the preferred communication channel for the report today? |
| Interprofessional collaboration and communication with patient | How have your relationships with the physician and the nurse evolved? We perceived that you have found a new place in your relationship with patients who request these collaborations, that you are happy, it motivates you and at the same time is sometimes a little complicated. We would like to bring this subject to the table and ask you what small steps are being taken to better structure these relationships? |
|  | Have you contacted the physician or the nurse in particular situations and for what reasons? Have you had to contact the usual patient pharmacist in these last 6 months and in what situations? |
| Facilitators | What are the new facilitators encountered in the last 6 months? |
| Satisfaction | What are your impressions now about the program and how it works? |
| Barriers | Conversely, what new barriers have you encountered since our previous focus group? The greatest facilitator was the inclusion of patients by the medical team. We perceived a difficulty in proposing the program to a patient, especially in the case of HIV patients. We also felt the need to create a relationship of trust with the patient before to be able to propose the program to him. How is it going now? Have you included patients in the last 6 months? What could help you be more comfortable with this complex relationship with HIV patients compared to others? |
| Patient selection criteria | What are the patient selection criteria?  Have you sometimes chosen not to offer the program to an eligible patient and for what reasons? |
| Positive or negative impact on patients | What impact do you think this program has on your patients? You told us it was too early to talk about the impact of the program on patients. What about now? Could you give us specific examples? Have there been negative impacts and if so, what? |
| Motivation for implementation and perceived utility | Have your motivations for implementing this program evolved over time? In your opinion, what are the skills and information you could provide to physicians/nurses? What is the relevance of this information according to you? |
| Suggestions for improvement | What would your suggestions be for improvement of the program and its practical progress?  In April, you told us that the interviews were not invoiced because only one drug was monitored. Is that still the case today? Have you considered monitoring other chronic medications for these patients? |

1. **Third focus groups with healthcare professionals**
   1. *With the physician and the nurse*

| **Outcomes** | **Open-ended questions** |
| --- | --- |
| Hospital’s adoption | How did the integration of the medical intern take place? Has he already referred patients? So far, the second infectious disease specialist has not yet taken part in this program. In your opinion, can this evolve? |
| Organisation | Previously, you raised the fact that your roles were not yet clearly defined in terms of practical organisation for this program. Have you been able to discuss this in the last 6 months? How did it evolve? |

- 1. *With pharmacists*

| **Outcomes** | **Open-ended questions** |
| --- | --- |
| Fidelity | In previous focus groups, we found that you all more or less adopted the program, each in your own way. It is a time-consuming process – you need to acquire experience to feel more comfortable delivering this program. Interviews are easier with time and when links are created with patients. But you have also dealt with patients who you have described as "complicated" or "complex" and compared them with "easy" patients. Since December, how have you worked with these "complex" patients? Has the experience of these last 6 months given you new ways to take care of these patients? (What would be the elements of discussion or intervention that could unblock these situations?) Have you faced other "complicated" situations than those already mentioned? Do "easy" patients remain easy over time or are there things that get more complex? |
|  | To continue, I would like a little better understanding of how the interviews with your patients begin. If you were in front of one of your patients right now, how would you start the interview? (Do you report the medication adherence electronic results directly to the patient?) Do you count pills in the electronic monitor at the beginning of the interview? If yes, could you describe the aim(s) of this? In some situations, do you think that the pill count could have brought something to the interview? Do you think the pill count could validate the results of the electronic monitor? |
|  | Some of you wrote your report by hand and then transcribed it into SISPha to send it to the physician. Is this still the case today? Have you considered writing the report directly in SISPha? |

- 1. *With all healthcare professionals*

| **Outcomes** | **Open-ended questions** |
| --- | --- |
| Evolution of patient inclusion | Physician/nurse/pharmacists: We are now seeing inclusions stagnate a little. Are you satisfied with the evolution of inclusions in the program? What do you think could increase these inclusions?  Physician/nurse: The reasons for inclusion were new treatment, a treatment switch and the prevention of medication adherence issues in patients with psychosocial problems to avoid an increase in viremia and the emergence of resistance. For you, it was to act before the problem occurs, and you mentioned it followed medication adherence in the same way as viremia and CD4. Do you agree with that? What do you expect from the pharmacist?  Pharmacists: What do you expect from the physician and the nurse during inclusion? |
| Refusal evolution | You mentioned that the most complicated patients, who are certainly those that would benefit the most from this program, often refused it. How has this thinking evolved now? How could these patients be included, according to you? Were there any reversals? (refusal becomes an inclusion) |
| Report | Several positive points were mentioned by the physician and the nurse concerning reports: - Report = starting point for discussion with the patient about medication adherence  - Highlight the situation without aggressive confrontation - discussion without intrusion - Different speech/approach if difficulties were highlighted in the report and strengthen the follow-up of patients with proven difficulties in medication adherence. Physician: Reading this report before the patient medical visit is integrated into routine activity. What are you looking for in a report? Information, material? What is important to you? What mechanism/process do you use to find the information? During the last focus group, there was a clear interest in receiving these reports before the medical visit. To what extent do you get these reports at each visit? Has there been a noticeable change in the frequency of sending reports? |
| Interprofessional collaboration – alignment of practices | In previous focus groups, you all noted that information given by the patient to the pharmacist, the physician and the nurse is complementary and that for this reason, communication channels between you are important. In the first year, we felt that these communication channels began to be developed but were not yet fully developed. In the last 6 months, how have these communication channels evolved? |
| General evolution | Do you have any remarks concerning the evolution in the implementation of this new program? |

1. **Fourth focus groups with all healthcare professionals**

| **Outcomes** | **Open-ended questions** |
| --- | --- |
| Satisfaction | It has been 2 years since the project was launched. What is your overall satisfaction with the implementation of this medication adherence program? How satisfied are you with patients? Have the objectives stated at the beginning of the project been achieved in relation to your activity and to patients? (Share the time needed for these patients between professionals, maintain good clinical results and catch patients before virological failure, bring additional support to the patient + safety expected of the physician and the nurse - interactions, treatment management) |
| Inclusions/ refusals | What has been the evolution of inclusions over these last 6 months? What was the involvement of the medical intern in this program? Has there been a change of medical intern during these last 6 months? In the last 6 months, have you encountered any new refusals? If so, what were the reasons? What is the evolution of inclusions concerning other pathologies? (Inclusion was considered as more complicated with HIV patients, but what about other pathologies?) |
| Follow-up of patients | During the last focus group, you explained to us that complex patients became easier over time and easy patients remained easy. Is this still the case today? How do interviews evolve over time (content, interventions)? Have you terminated some patient follow-ups, and if so, how (organised by the pharmacist, proposed by the physician, desired by the patient)? |
| Interprofessional collaboration | Six months ago, you talked about setting up a more bi-directional communication between you, with overall feedback on the clinical results and the return on the positive results of the patients. How has this evolved in the last 6 months? |
| Maintenance | Over these 2 years, we have organised 4 meetings. What do you think about these meetings? What did they bring to you? What do you think would drive the expansion of this program to other pathologies within this hospital? In contrast, what would be the barriers? What elements would you need to sustain this program over the long term? Would external support still be needed? |

1. **Patient interview grid**

| **Outcomes** | **Open-ended questions** |
| --- | --- |
| Motivation | What are the reasons that motivated you to participate in this program? How have your motivations evolved over time? |
| Perceived utility of the program | Do you think this program is useful for you? Could you describe the reasons? Has this usefulness evolved over time? |
| Integration of the electronic monitor into daily life | How did you integrate the electronic monitor into your daily life? |
| Factors that facilitated participation/follow-up | What facilitated your participation and follow-up? (Elements, events, the way it was proposed to you or a particular moment) How would you have reacted if your pharmacist had offered you this program? |
| Barriers | What problems have you encountered since you started participating in this program? (Time, travel, new interlocutors, or others) |
| Relationship with healthcare professionals | What are your relationships with the health professionals (physician/nurse and pharmacist) in this program? |
| Positive or negative impact | What did you get out of this program (positive and/or negative)? What else has this program brought you? To what extent did the program help you talk about medication intake with your physician/nurse/pharmacist? |
| Satisfaction | What are your impressions of the program and how it works? (Regarding your initial expectations, duration of the interview, frequency of the interviews, practical organisation, appropriate place for the interview, or others) What were your expectations of this program? |
| Suggestions for improvement | What would be your suggestions for improvement of program and its practical progress? |
