# Supplementary material for: Implementation study of an interprofessional medication adherence program for HIV patients in Switzerland: quantitative and qualitative implementation results
Source: BMC Health Serv Res. 2018 Nov 20;18:874. doi: 10.1186/s12913-018-3641-5 (PMC6247756; doi:10.1186/s12913-018-3641-5)
Supplement: Supplementary file 2 — Content of organised meetings and focus groups during the operation phase. Description of data: Description of topics covered during meetings and focus groups with health care professionals. (DOCX 20 kb) [file 12913_2018_3641_MOESM2_ESM.docx]

**Additional file 2 - Content of organised meetings and focus groups during the operation phase**

| Timing | Covered topics during focus groups (FG) |
| --- | --- |
| First meeting (April 2015) | One FG with the physician and the nurse and one FG with the five pharmacists.   - motivation for and perceived utility of the program - integration into the healthcare provider routine activity - practical organisation and needed adaptations - barriers and facilitators - interprofessional collaboration - perceived impact of the program on patients - satisfaction |
| Second meeting (November 2015) | One FG with the physician and the nurse, one FG with four pharmacists and one separated interview with one missing pharmacist (Pharm 5). *Covered topics:*   - evolution of topics discussed during the previous FG |
| Third meeting (May 2016) | One FG with four pharmacists and one separated interview with one missing pharmacist (Pharm 3).   - management and follow-up of complex patients - writing and sharing the adherence reports with the physician and nurse, and validation of electronic monitor results   One FG with the physician, the nurse and four pharmacists.   - monitoring of inclusion and refusal reasons - added value of the adherence report written by pharmacist - communication and exchange of the PMU experience with complex patients; “Patients who do not admit ART omission”, “ART treatment switch”, “influence of life events on medication adherence” and “Patient who refuse to use an electronic monitor”   One FG with the physician and the nurse.   - inclusion of the intern in the program |
| Fourth meeting (December 2016) | One FG with the physician, the nurse and two pharmacists (Pharm 4 and 5) and one separated FG with two missing pharmacists (Pharm 1 and 2).   - overall satisfaction - evolution of patient inclusion and follow-up - sustainability of the program over time - exchange of the PMU experience about adherence reports, and information transmission between the pharmacist and the physician/ nurse |
