# Supplementary material for: Implementation study of an interprofessional medication adherence program for HIV patients in Switzerland: quantitative and qualitative implementation results
Source: BMC Health Serv Res. 2018 Nov 20;18:874. doi: 10.1186/s12913-018-3641-5 (PMC6247756; doi:10.1186/s12913-018-3641-5)
Supplement: Supplementary file 3 — Themes inferred during the qualitative analyses and associated sentences. Description of data: Themes defined by researchers and associated sentences classified by dimensions and measured outcomes. (DOCX 54 kb) [file 12913_2018_3641_MOESM3_ESM.docx]

**Additional file 3 – Themes inferred during the qualitative analyses and associated sentences**

| Re-AIM | Outcomes | Themes | Phrases |
| --- | --- | --- | --- |
| Reach | Facilitators for reaching patients (according to HCPs) | Inclusion by the physician and the nurse | *Pharm2: "When they come from the hospital, the physician contacts me and tells me ‘I’ve a patient for you’ then the upstream work is already done and the patient know that we are waiting for him, that we already worked on his file. It’s not the same thing.”*  *Pharm3: "We cannot arrive just like that with a proposal of interview, patients wonder what is going on. When the physician proposes it, obviously it happens right away."* |
|  |  | Inclusion of naïve HIV patients | *Nurse: "It is much easier when we start the program at the beginning of the ART-therapy. It is more complicated to present the program to patients who have already been followed for a long time."*  *Physician: “It was his first treatment. I think it now seems natural for him to function like that… we benefit from the overall effect of ‘novelty’.”* |
|  | Facilitators for reaching patients (according to patients) | Appropriate description of the program and reassuring information received | *Pat3: "All the explanations they gave me reassured me… it allowed me to want to participate"* |
|  | Barriers to reaching patients | Reasons for refusal sometimes unclear – possible escape for some complex patients | *Physician: "Patients said that the program is good, that the opportunity to get an additional person around them is good but for reasons that are sometimes still intangible, they don’t want to start right away… There is often an argument that may be assembled somewhat as a protection. ‘I don’t want to change pharmacy’ is the argument behind which they take refuge. It maybe hides the fact that they don’t really want someone to get interested in it [their behaviour]."*  *Nurse: "We observe the same thing during the consultation. These patients, who have great adherence difficulties, also had a meeting with me to discuss adherence (…) but they also didn’t come to me."* |
|  |  | HIV-related difficulties (psychosocial issues with stigmatisation, denial and need for a high confidentiality level) | *Pharm2: "We feel that it remains a taboo disease even now.”*  *Nurse: “They know different technicians and say ‘Oh no, I don’t want to go there because one of my friends is there,’ so it is true that it is often a brake, they would like to know nobody [among the pharmacy staff].”*  *Physician: “It is focused on the secret, about not divulging. They are going to get their medicine quietly away from home, they are very focused on the concept of hidden disease.”* |
|  |  | Limited number of trained pharmacies | *Physician: “We are a little bit limited by the fact that only some pharmacies are available to deliver this program.”* |
|  |  | HIV = small population base | *Physician: “We think about the program for all new patients, but again we do not have a new patient every 5 minutes … it’s true that initial patients were all included and in the end we have an initial pool that we expected for this project. After all, the program is now a routine activity, but it seems logical that inclusion stagnates a little quantitatively.”* |
|  |  | Non-inclusion by pharmacists – difficulties in identifying target patients | *Pharm1: "In the end, how do you know they need it?"* |
|  |  | Non-inclusion by pharmacists – proposing the program at the counter 🡪 breaching confidentiality | *Pharm2: "It’s difficult because it’s true that it is almost necessary to take the patient aside to present [the program] because if we propose it at the counter in front of other people, we do not know how he may feel … it is just my point of view but for us [pharmacists], it is actually easier to propose this to a diabetic patient than to an HIV-patient."*  *Pharm5: "HIV patients come to the pharmacy only for their ART-therapy. It’s a little bit more complicated to propose an interview while patients are trying to be as discreet as possible … adherence may be more easily addressed because the disease [diabetes] is much more common and less stigmatized so we will probably talk more easily about it at the counter … but actually I think that barriers are the same after the proposal [same reasons for refusals whatever the type of disease]."* |
|  |  | Non-inclusion by pharmacists – difficulties in acknowledging the added value and benefits of their program /afraid to propose a “paid service” | *Pharm4: "The pharmacist does not know how to sell himself… when we have to push ourselves forward, show our competencies, we are not at ease with it and we are afraid to ask patients to pay."* |
| Adoption | Motivation for integrating the program | Hospital – initiating event: local experience with a non-adherent patient | *Physician: “I started a little to do this in collaboration with the nurse who had training in therapeutic education … in an informal way and then we started formalizing it through this project … it’s with this type of patient, they take time that is disproportionate in comparison to the average HIV patients. We also sought help because we don’t have hours to spend on these patients, we try to get more support around these patients with other people, pharmacists in particular … just me and the nurse, it is impossible for all patients in any case.”* |
|  |  | Pharmacists – future of the profession/opportunity to prove their added value | *Pharm1: "This is the future of our pharmacies."*  *Pharm2: “This project is useful for the added value that it brings to our profession.”*  *Pharm3 "It’s part of our mission."*  *Pharm4: "It emphasizes the pharmacist’s role, projects in collaboration with the physician, we cannot stand out of this circle."*  *Pharm5: “Being recognized, seeing the interest it has for health and at the economic level.”* |
|  |  | Pharmacists – springboard/opportunity to include patients and get more experience in medication adherence | *Pharm4: “…This study allowed us a little bit to introduce ourselves into this [medication adherence program], to practice with these electronic pillboxes beyond the study itself, in order to be able to introduce the SISPha program [diabetes] later.”* |
|  |  | HCPs – support complex patients | *Physician: “The pharmacist becomes another HCP the patient can refer to in his/her close community, this pharmacist has access to the disease of the patient, he/she has a vision, the opportunity to bring additional support, in proximity to the patient, this is of interest.”*  *Nurse: “… I also think that it’s also important for them, even patients who adhere well, to be able to talk about what they live, their difficulty in taking a drug, even if everything seems to be going well.”* |
|  |  | HCPs – develop interprofessional collaborations | *Physician: “We try to diversify healthcare professionals who gravitate around these patients to try to have the input and the specificity of everyone.”*  *Nurse: “Yeah, interdisciplinarity plays a role, it’s clear, also the knowledge of the pharmacist.”*  *Pharm2: “This link, yes, I agree with my colleague (Pharm1: yes), it is important in this disease that we intervene in collaboration, multidisciplinarity, but we also have our role to play, we have to make this connection with the patient.”*  *Pharm3: “Place us in partnership with the patient and the physician.”* |
|  | Perceived utility | Improvements in clinical results | *Physician: "Typically, the patient who was the initiating trigger of this project, we saw that her results improved by investing a little more interest in her life, in the factors that influenced her adherence … We see a benefit to supporting medication adherence.”* |
|  |  | Development of trust with the pharmacist, who became a reference person | *Pharm1: “It’s a trust relationship that is established.”*  *Pharm4: “We feel in many patients that it makes one additional person to whom they can talk about their disease.”* |
|  |  | Pharmacists = contact point for holding back patient loss to follow-up | *Physician: "There are more professionals around patients and less chance of getting lost … the pharmacist also become an additional reference person."* |
|  |  | Electronic pillboxes reassure patients | *Pharm1: "It's really a crutch for her."*  *Pharm2: "When the patient has evidence that he is taking his treatment properly, it strengthens and motivates him."* |
| Implementation | Time needed | Took more time at the beginning of the implementation process – does not require substantial additional time work | *Physician: "It required some efforts, habits to take up concretely but now, personally, I cannot say that it takes a lot of time … It is well integrated I think now. We do it routinely without necessarily requiring major work."*  *Nurse: "It’s clear that it’s time that we lose at the beginning and that we gain after.”* |
|  | Fidelity – interview with the pharmacist | Active-listening skills | *Pharm1: “We talk more about his life, we also talk about his disease at the beginning, how things went with his family, with his parents etcetera.”*  *Pharm2 “The important thing is that the patient feels that he is followed, that he feels supported, that he is accompanied, that if he has a problem, we listen to him and we try to help.”*  *Pharm3: “My only trick is to try to dig a little with open questions to see if there really is something special then I look out for the little ‘everything is fine BUT’.”*  *Pat4: "The medication intake is now automatic, so the fact that there are these questions, someone who is interested, we put our daily gestures into words … well, it obliges me to speak, reflect on how I function.”*  *Pat7 "We have to check my pillbox together, how I have taken [my medication], whether the schedule is good or not or if there are things I need to improve. I have to discuss how I felt throughout the month."* |
|  |  | Progress at the patient’s rhythm | *Pharm2: “I would say that as we see her every week. It’s great. I find it progressively creates a trust relationship then the person, when we conduct the interview, will talk more easily and confide much faster.”*  *Pharm3: “We are still in the approach phase, which means that I don’t know, they don’t trust me yet or they don’t want to confide in me. It will come later.”*  *Pat7 "He did everything for me to be able to trust him because I was scared at the beginning.”* |
|  |  | Look for solutions in collaboration with patients | *Pharm2: “It is the aim that she manages to integrate it into a ritual, so it is necessary that I work with her, I think, to try to find a solution by herself to integrate it in her daily life.”*  *Pat7 "I can take initiatives and propose them, I do not just have to wait for everything from the physician because the physician said this or the pharmacist said that.”* |
|  |  | Value patients’ self-management achievements | *Pharm1: “I congratulate him.”*  *Pharm4: “This is essential.” [talking about valorisation]*  *Pharm5: “Each time she is congratulated, it’s perfect.”*  *Pat4: “The fact that there are several people who say ‘it's good, continue in the same way’ is encouraging, I actually do not feel alone."* |
|  |  | Concerns about tackling some adherence issues with patients | *Pharm1: “It is difficult to speak with him because he doesn’t speak too much, and even when I ask him open questions, it’s difficult because he tells me what he wants to tell me, that’s it. I do not dare too much … I didn’t ask questions because, yes, I didn’t want to, I think it was a little bit intrusive.”* |
|  |  | Patients would perceive the intervention as ‘policing’ | *Pharm4: “This is essential. If we use this method of control and the control shows that things are done well, if we do not value it, we cannot control it and then it becomes policing and we intervene when it is negative and it is no longer a help.”* |
|  | Fidelity – use of the electronic monitor (EM) | One pharmacist did not use the electronic monitor 🡪 neither the patients nor the pharmacists were at ease with the monitoring and perceived it as a form of control | *Pharm3: “Both [patients] said they took it very regularly. We checked with the electronic pillbox, it was very regular. I told them ‘listen, stop the electronic pillbox since you take it as well as that, but let’s keep up contact’. They both agreed … I was trying to see what their need was rather than adapt them to some protocol or other. When the two said to me ’You see, why are we continuing to do the graphs? Because you see that we take it well’ … so they did not want to show the graph to the physician.”* |
|  | Fidelity – reports | Reports not sent systematically to the physician and the nurse | *Physician: “They don’t willingly give spontaneous feedback.”*  *Pharm4: “If there is nothing new, I save them the opening and reading time.”*  *Pharm1: “I think it’s good to send them a small report from time to time between the 6 months to tell them that the patient still has good adherence. It can be useful too.”*  *Pharm3: “What I wanted to do is at the end of this year was to do an interview with the two patients and then send an overall report for this year to the physician, because I have not made other reports since there was not too much to say. I didn’t want to send them reports saying that everything is going well.”* |
|  |  | Concerns about the relevance of the transmitted information | *Pharm1: “I think this is a minimum if as a pharmacist we want to get into these talks. We must be credible.”*  *Pharm4: “For me, we have to work, work, work to prove to them that pharmacists are not useless.”* |
|  |  | Concerns about disturbing the physician with too many reports and increasing his workload | *Physician: “Apparently, what I felt, is that they had a few scruples about sending us reports so as not to flood us with paper but [laughing] on the other hand, we are glad to receive it.”*  *Pharm4: “If I don’t have relevant elements, which I consider relevant, maybe I’m wrong, I say to myself ’Why disturb him?’. If everything is going well, if the graphs are good, if there is no complaint of side effects, if there is no problem, I will not drown him under paperwork.”* |
|  | Facilitators | Involvement of the physician and the nurse in the program in including patients and acknowledging the pharmacist’s role | *Pharm2: “I think it is really a big facilitator if the physician proposed the program.” … "We know that the physician is favourable to our cause."*  *Pharm5: "We know that the physician is open, what they want from us, they trust us. Therefore, it is much easier to continue in this way."* |
|  |  | Resources put in place for the program (research support, web platform and training courses) | *Nurse: “We would never have done that without you [talking about research team].”*  *Pharm2: “The tools that the platform offers are completely adapted.”* |
|  |  | Regular meetings between stakeholders – drive the project forwards (rewarding and stimulating, get feedback from each other and exchange experience, know each other’s expectations and facilitate communication in practice) | *Physician: "These meetings were useful to move the project forward, for purpose of maintenance, to ensure that everybody get something out of it. We managed to improve communication, discussed what we can do better. I think it was valuable to meet for that."*  *Pharm1: “These meetings were rewarding… It was actually motivating."*  *Pharm2: "It [meetings] was very positive because I was not there at the beginning but also because I felt less alone on the project… It was good to be able to exchange with others, to also have the experiences of other stakeholders. Then also to have contact with the physician and the nurse … It is much easier to approach them in case of problems. We saw them, we know them.”*  *Pharm4: "It’s easier to call a physician we know… it is very useful, first to have the experiences of the colleagues who are doing the same project, second to be able to exchange with others in term of interdisciplinarity; to know what the physician needs, what he would like, what we could like, what we need.”*  *Pharm5: "It is really interesting to be able to meet each other, to exchange between us and to know who you are addressing, more than an email or a name… yes exchange is easier.”* |
|  |  | Small medical team facilitated internal communication | *Physician: “For inclusion, I think we talk anyway, we cross frequently… we have not really formalized things because there are two of us, now we have a third person with the medical intern, but I think we benefited from being a small team, that is, we communicate quite easily.”*  *Nurse: “It’s the advantage of small teams, it is clear that we can talk easily.”* |
|  | Barriers | Lack of time related to lack of resources | *Physician: “Sometimes, there is also a time problem during the medical visit. Patients, for whom 30 minutes of consultation are planned, arrive with a lot of somatic problems and we do not have always the time to have such long discussions, but in general, it postpones the proposal.”*  *Nurse: "We are nevertheless limited in staff too."*  *Pharm1: "When you are the only pharmacist in the pharmacy, effectively, it takes 30 minutes to do an interview; it’s sometimes a lot."*  *Pharm3: "I was there part-time and we are still understaffed for what we do."* |
|  |  | Low adoption by the management because of lack of sufficient financial incentives | *Pharm1: "We have a delivery problem too, we do not charge anything" … Pharm2: "It's clear that yeah, our bosses behind us are not too much for it"*  *Pharm4: "This is the future but on the other hand it remains technically unprofitable with patients."* |
|  |  | Lack of team uptake | *Pharm1: “The other pharmacists are able to do it but do not … actually I’m all alone.”*  *Pharm5: "For pharmacy technicians, this is the job of the pharmacist because we have few patients and they have no demand for this but just ask them to be motivated to propose the program. They consider that it’s not their job.”* |
|  | Barriers and facilitators for patients | Time required for interviews acceptable | *Pat5: “I see the program positively. I will take the time. I will integrate it in my personal program without it being a problem for me.”*  *Pat6: “It’s clear that I had to devote one or two times my time to this but I accepted it so I did it.”* |
|  |  | Organisation of interviews at the pharmacy | *Pat7: “It’s often difficult to find time to collect my medicine but the pharmacist always ensures that I have my medicine, whatever his tight schedule or my tight schedule… so we organize ourselves in such a way, so that on weekends, he can find time to also be available and come to collect my medicine.”* |
|  | Development of interprofessional collaboration | Synergy and complementarity of the information given by the patient | *Physician: “It generated synergy to make him understand how to take his medicine and have more professionals that say the same thing, I think it was useful.” … “They don’t talk necessarily about the same things and information is often complementary.”* |
|  |  | Report useful during the medical visit (open the discussion with patients and integrate the information into the clinical decision) | *Physician: “It’s a way to open the discussion with the patient, show the adherence report, congratulate patients; even if it is positive and it remains positive, it can be useful for us … it is the pretext to discuss … and then when it’s not so good, to discuss openly how it happened and address what they tell us, in the end a pretext for the discussion about adherence.”* |
|  |  | Strengthen patient safety regarding interaction management, OTCs and regular treatment delivery | *Nurse: “There is also this interesting aspect of relief, of telling ourselves that the pharmacist will be interested in knowing when the patient no longer has treatment.”*  *Physician: “We are always reassured when patients go to the same pharmacy for all their treatments, there is a check-up that is done… I think we are happy to have all the checking on interaction.”* |
|  |  | Opportunity to share responsibilities for complex patients | *Physician: “I think that sharing responsibilities, having more professionals around these complex patients, at least temporarily complex, also allows to preserve ourselves a little bit.”* |
|  |  | Opportunity to decrease the risk of patient loss to follow-up | *Physician: “Probably that sometimes it saved us time or even allowed us to keep patients that may have disappeared from the consultation … There are more people gravitating around and therefore less chance that we will lose them … the pharmacist in this case reminds her from time to time to come and see us for blood tests, so it also works in that way.”* |
| Sustainability | Inclusion into routine activity | Hospital – structural change 🡪 allowed the integration of the new intern in the program + inclusion of the program into the local hospital guidelines | *Physician: “It worked out naturally. The nurse did a guidelines book for all new medical interns about all important things they need to know about HIV and viral hepatitis, an introductory guide for a physician who has no specific training. Since then, we have introduced this notion for all new treatments so the arriving physician does this in a somewhat routine manner.”* |
|  |  | One pharmacist – inclusion of a second pharmacist – team organisation with a shared calendar to organise interviews | *Pharm2: “We have a calendar in which we can really see all the interviews. We know who comes, when, at what time, who follows the patient … afterwards, we also know that if one of us is not there, we can take turns because, in the end, we do the same for all the patients.”* |
|  | HCPs’ satisfaction | Ideal collaboration – professionally satisfying activity – improve patient support | *Physician: "We are glad to be able to offer an additional program that was not available before and that I think has already proven its worth … it is professionally satisfying to be able to better take care of patients.”*  *Nurse: "They know you are there [pharmacists] so they may go back with their pillbox* *easily. We know that medication adherence fluctuates, a period when everything goes well, so I think they have an important anchor … I’m also very happy to see that patients are satisfied.”*  *Pharm1: "Personally, what I like with the physician and the nurse here, is that we have feedback… it is a mutual enrichment.”*  *Pharm3: “We work better by exchanging patient information.”* |
|  |  | For pharmacists – instructive project – opened doors to more pathologies | *Pharm1: “It’s a beautiful gateway, it motivated us, it’s true, to launch ourselves.”*  *Pharm2: “It’s good because it trained us. It has forced us to enter into this and then it opens the way for other things. Now there is the diabetes project … now the infectious disease department also send us patients who take therapies for hepatitis … it is slowly starting to open up a little bit to other things.”* |
|  | Patient satisfaction | Positive perception of the program | *Pat3: "I think it’s a good thing that this was able to be put in place, really, to help people in the same situation as us. I appreciate this program. I take it in the right way for me and I congratulate the people who were able to get it started.”*  *Pat7: "It’s a positive case, also in the sense that you are truly supported at all levels."*  *Pat8: "maximum satisfaction"* |
|  |  | Development of a relation of trust with HCPs | *Pat7: “He did everything so that I could trust him because I was scared at the beginning.”*  *Pat8: “I’m confident with the physician, the pharmacist and the nurse.”* |
|  |  | Additional social support – reassured them | *Pat4: “We do not feel alone.”*  *Pat5: “To feel supported, yes I think it is to be reassured, the follow-up and everything.”*  *Pat7: "First, when I discovered this health status, it really shook me and I had insomnia, which was also addressed by the collaboration between the physician and the pharmacist … it was a very strong support, it happened during a psychologically unstable period.”* |
|  |  | Benefit from a reference person at the pharmacy | *Pat3: "Now that I see the same pharmacist, I am more motivated … I have more trust because I am used to that person."*  *Pat8: “The person already knows how I am and how I feel with the treatment. If it changes, I need to explain everything again.”* |
|  |  | New opportunity to discuss their medication | *Pat4: "I was not used to speaking so much because I never really had the opportunity to do so."*  *Pat5: "I like it because for a little while now if I have questions about another medication that I am hesitating to take or things like that, it helps me”* |
|  |  | Support in the treatment management | *Pat3: "The program helped me a lot, reassured me in my disease management and in my medicine management.”*  *Pat4: “The medication intake, now it is automatic, so the fact that there are these questions, somebody who is interested in it, we put words to our everyday acts… it obliges me inevitably to speak, to think about how I do things.”*  *Pat7 "What I found really good is the time to talk about everything in detail, that I can understand. Because, for example, there are side effects I didn’t understand ... the side effects were nausea, gastric lift, the trick was to know how to improve by looking a little at how I feed myself.”* |
|  |  | Feedback from professionals encouraging | *Pat4: “The fact that there are several people who say ‘it's good, continue in the same way’, it is encouraging, I actually do not feel alone."* |
|  |  | Electronic monitors – reassuring + prevention of omissions | *Pat1: "I am not necessarily very consistent in terms of medication intake, in the end I never took any until I had to take these ... it’s true that sometimes when I come back home, it’s a reflex to check every time if there is a little 1 or 0 … it reassures me since I know I’m easily an ‘airhead’."*  *Pat3: “I really appreciate this thing [the electronic monitor] because with it, we are sure we took the medicine every time. Because there are times when sometimes during the day I don’t know if I took the pill or not. Then with this, I just need to look at the pillbox and I know if I took it, so I’m sure.”* |

**Notes**: Phrases correspond to verbatim transcription from French to English. HCPs = healthcare professionals (pharmacists, the physician and the nurse)
