# Supplementary material for: Implementation study of an interprofessional medication adherence program for HIV patients in Switzerland: quantitative and qualitative implementation results
Source: BMC Health Serv Res. 2018 Nov 20;18:874. doi: 10.1186/s12913-018-3641-5 (PMC6247756; doi:10.1186/s12913-018-3641-5)
Supplement: Supplementary file 4 — Flow diagram of the IMAP as implemented at the hospital and in community pharmacies. Description of data: Implemented activity at the hospital and in community pharmacies as described by each included health care professional 2 years after implementation start (1 physician, 1 nurse, 4 pharmacists) (compiled version). (DOCX 73 kb) [file 12913_2018_3641_MOESM4_ESM.docx]

**Additional file 4 – Flow diagram of the IMAP as implemented at the hospital and in community pharmacies**

- New patients
- Treatment switch
- Prevention (in case of psychosocial issues)

**Email/call to the pharmacist:**

- Reason of inclusion
- Summary of the patient’s situation (medical/psychosocial)
- Goal(s) to achieve
- Possible other drugs to be monitored

**6. End of follow-up**

- Read the report
- Discuss the results with the patient
- Give feedback to the pharmacist concerning clinical data by sending a copy of the transmission letter intended to the general practitioner

Complete the document for the patient proposal

**3-5. Interview [pharmacist]**

**Selection criteria**

**1. Proposal [physician-nurse]**

**2. Inclusion**

**[physician-nurse]**

**4. Reception of Adherence Report [physician-nurse]**

The patient agreed

Subsequent program proposal in case of poor clinical outcomes

The patient refused

a. Flow diagram of the activity at the hospital

Note: The dotted arrow means that the patient can enter the program again according to individual needs

- Medication adherence results, summary of the points discussed and of the defined goal(s)
- Contact with the physician in case of identified problems

**3-6. Report to the physician – nurse [pharmacist]**

- Presentation of the program and of the electronic monitor
- Signature of the consent form
- Drug history
- Additional information on treatment, disease and adherence
- (Validation of electronic data since the last interview, omissions, pocket doses)
- Read the electronic monitor and present the results to the patient
- Discuss the drug intake rituals, the side effects, the patient’s motivation and psychosocial factors that can affect adherence
- Value the patient and support him/her in the search for solutions
- Summarize important points discussed during the interview
- (Set a goal for the next interview)
- Fill medication into the electronic bottles
- (Fix the next appointment)

**1. Proposal by the physician-nurse**

- Reasons for inclusion
- Goal(s) to achieve
- Contact preference and desired frequency for reports
- Drugs to monitor
- Retrieve the electronic monitor(s)
- Congratulate the patient for one’s involvement in the program
- Determine goal(s) related to adherence beyond the program
- Inform the patient about possible re-entry into IMAP in the event of new problematic situations

**4. Medical visit [physician – nurse]**

**7. Last interview [pharmacist]**

**(1.1. Contact with the physician – nurse)**

**2. Inclusion interview [pharmacist]**

**5. Follow-up interview [pharmacist]**

**(1. Proposal of follow-up by the pharmacist)**

- List of patients by disease (e.g. infectiology)
- List of patients by physician
- Note in the patient file

**(0.1 Identification of target patients)**

- Presentation of the program
- List of eligible patients to validate with physician
- Establishing collaboration

**(0.2. Information letter/call to the physician)**

- Presentation of the program to the patient
- Distribution of the information leaflet

Agreed

- Notification in the patient file
- Subsequent program proposal

Refused

b. Flow diagram of the activity in community pharmacies

Note: the information in brackets represents activities that are not systematically performed in all pharmacies (= adaptations among pharmacies)
